# *sli* is required for proper morphology and migration of sensory neurons in the *Drosophila* PNS

**DOI:** 10.1186/s13064-019-0135-z

**Published:** 2019-10-24

**Authors:** Madison Gonsior, Afshan Ismat

**Affiliations:** Department of Biology, University of St. Thomas, Saint Paul, MN 55104 USA

**Keywords:** Sli, Chordotonal neurons, PNS, Drosophila

## Abstract

Neurons and glial cells coordinate with each other in many different aspects of nervous system development. Both types of cells are receiving multiple guidance cues to guide the neurons and glial cells to their proper final position. The lateral chordotonal organs (lch5) of the Drosophila peripheral nervous system (PNS) are composed of five sensory neurons surrounded by four different glial cells, scolopale cells, cap cells, attachment cells and ligament cells. During embryogenesis, the lch5 neurons go through a rotation and ventral migration to reach their final position in the lateral region of the abdomen. We show here that the extracellular ligand *sli* is required for the proper ventral migration and morphology of the lch5 neurons. We further show that mutations in the Sli receptors Robo and Robo2 also display similar defects as loss of *sli*, suggesting a role for Slit-Robo signaling in lch5 migration and positioning. Additionally, we demonstrate that the scolopale, cap and attachment cells follow the mis-migrated lch5 neurons in *sli* mutants, while the ventral stretching of the ligament cells seems to be independent of the lch5 neurons. This study sheds light on the role of Slit-Robo signaling in sensory neuron development.

## Introduction

The nervous system is made up of neurons and glial cells. Proper development of the nervous system requires coordination between these two cell types. It is known that glial cells ensheath axons and function to drive nerve formation, provide trophic support for neuronal survival, provide guidance during axon pathfinding, and modulate dendrite morphology [1–4]. Even with all this information on the multitude of glial cell functions, we still have much to learn about how specific glial cells coordinate with neurons in the formation of the nervous system.

The fruit fly *Drosophila melanogaster* is a well-known model system for studying many of the fundamental aspects of neural development, including neuron-glia interactions [1–4], and the mechanisms and signaling pathways necessary for axon guidance [5–7]. For example, the Slit-Robo signaling pathways was found to be required for proper crossing of commissural axons in the central nervous system (CNS) in Drosophila [8–12]. In this study, we are examining more closely the role of *slit* (*sli*), and possible Slit-Robo signaling in the embryonic peripheral nervous system (PNS).

**Fig. 1:**
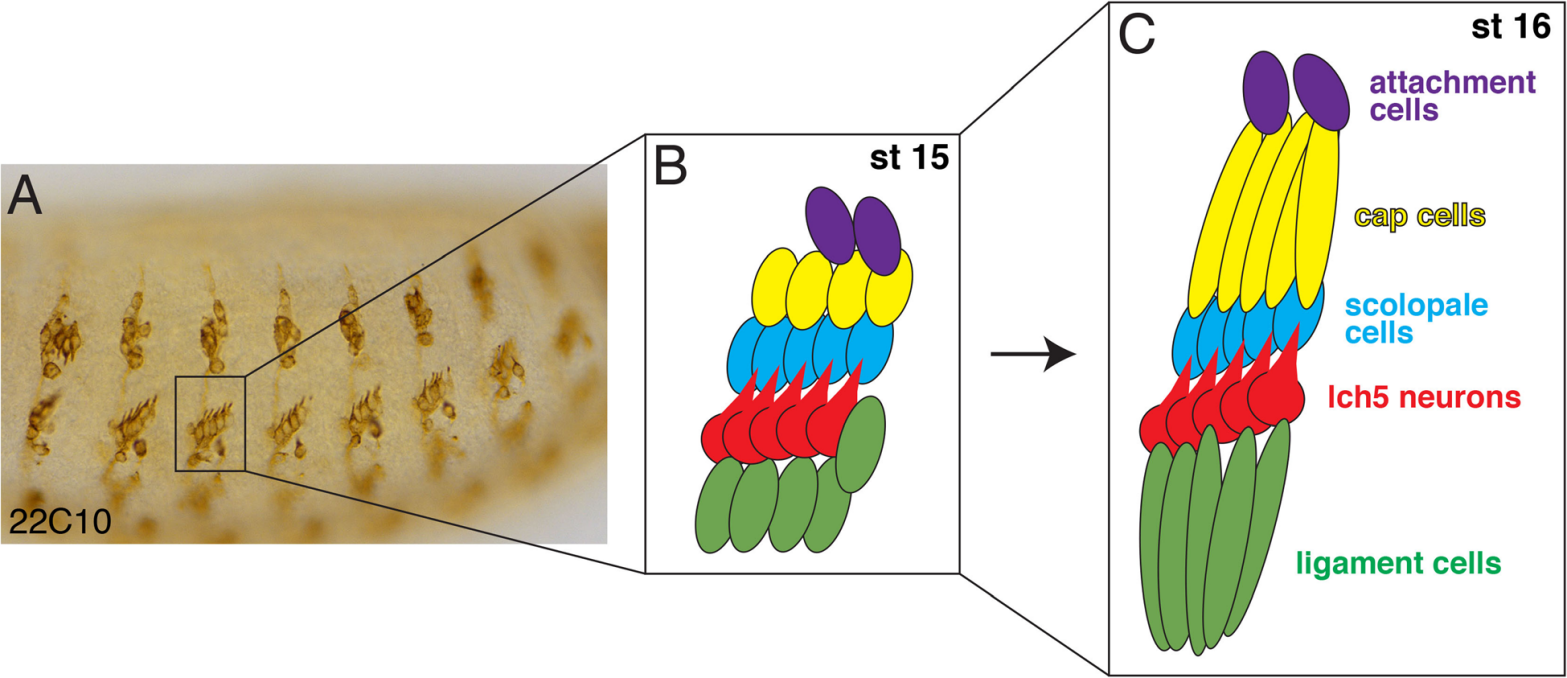
lch5 chordotonal neurons are surrounded by several types of support cells. **a**. Stage 16 wild-type (WT) embryo labeled with anti-22C10 to mark all PNS sensory neurons. The embryo is oriented anterior to the left, posterior to the right, dorsal to the top and ventral to the bottom. **b**. Cartoon depiction of one cluster of abdominal lch5 chordotonal organs (boxed region in A) at stage 15 showing the lch5 neurons in red, ligament cells in green, scolopale cells in blue, cap cells in yellow and attachment cells in purple. **c**. Cartoon depiction of the abdominal lch5 chordotonal organs at stage 16. The cap cells (yellow) have stretched dorsally and the ligament cells have stretched ventrally

The embryonic PNS of *Drosophila melanogaster* is composed of different types of sensory neurons, which are divided into Type I - neurons with single dendrites, and Type II - multi-dendritic neurons. Type I neurons are divided further into four clusters, dorsal (d), lateral (l), ventral’ (v’), and ventral (v), according to their final position along the dorsal-ventral axis of the embryo. The lateral chordotonal (lch5) neurons are a group of five Type I mechanosensory neurons that sense stretch and vibration [13–16]. There is one group of lch5 neurons in each of seven abdominal segments of the Drosophila embryo [14, 17]. The precursors of the lch5 neurons initiate in a dorsal position in the embryo at stage 12 and migrate ventrally to their final lateral position at stage 15 (Fig. 1A, B) [13, 15, 17–19]. By stage 15 these neurons have a very distinctive morphology, which includes neuron shape, direction of dendrites and spacing of individual neurons relative to each other (Fig. 1A, B, red cells). The shape of each of the five neurons in the lch5 cluster has a teardrop outline with the single dendrite pointing in a dorsal-posterior direction (Fig. 1A, B, red cells). These chordotonal neurons are surrounded by four groups of secondary (glial) support cells, scolopale cells, cap cells and attachment cells that are dorsal to the neurons, and ligament cells that are ventral to the neurons (Fig. 1B, C) [18, 20–22]. The lch5 neurons plus their support cells coalesce into one lateral chordotonal organ (lch5 organ). On the dorsal side, the cap cells are connected to the ectoderm by attachment cells [18, 22]. The scolopale cells surround the tip of the dendrite which may interact with migratory cues along the pathway [17, 20]. On the ventral side, the ligament cells stretch ventrally to attach the lch5 organ to the ectoderm (Fig. 1C) [17, 18, 22].

Unlike most of the neurons in the PNS, the lch5 neurons go through a rotation and migration during embryogenesis. Rotation and migration both occur after stage 12 with rotation followed by migration [17, 18]. Prior to stage 12, the dendrites of all thoracic and abdominal chordotonal neurons, including the lch5 neurons, face ventrally. After stage 12, the abdominal chordotonal neurons rotate until the dendrites face dorsal posteriorly [17, 18]. Although the exact mechanism of this rotation and migration is not known, a few different mutations have displayed lch5 migration and rotation defects. For example, it has been shown that Slit-Robo signaling does affect these two processes. Specifically, it was mentioned, but never shown, that in the absence of the extracellular ligand *sli*, the cell bodies of lch5 neurons either stay dorsally located or their dendrites are aberrantly oriented. The same defect is observed in a double mutant of the *sli* receptors *robo* and *robo2* [19]. Likewise, the Robo receptor is expressed at the tips of lch5 dendrites while Robo2 is expressed along the entire lch5 dendrites in abdominal segments [17]. In the thoracic region, Robo2 receptor expressed in the visceral mesoderm binds Slit and presents Slit to Robo receptors expressed on thoracic chordotonal neurons, thereby preventing migration of the thoracic chordotonal organs [17]. Additionally, loss of the transcription factor *ventral veinless* (*vvl*) results in a failure of the lch5 chordotonal neurons to migrate ventrally, similar to that seen in the absence of *sli* [18, 23]. The question we are asking is what role does Slit-Robo signaling play in lch5 neuronal migration and morphology.

In this study, we have sought to shed further light on the role of *sli* on migration and final morphology of the lch5 cluster. We show here that *sli* is necessary for the ventral migration, final positioning, and morphology of the lch5 chordotonal neurons in the Drosophila PNS. In addition, we show that the absence of either *robo* or *robo2* display similar, albeit less severe, defects. Further, we show that, in the absence of *sli*, the scolopale cells, cap cells and attachment cells follow the mis- or non-migrated neurons, whereas the ligament cells continue stretching ventrally in the absence of *sli*. Thus, we have shown that Slit-Robo signaling is important for yet another piece of neural development.

## Materials and methods

### Drosophila strains

The following Drosophila strains were used in this study: Canton S (CS) as the wild-type (WT) control, *sli*^*2*^ (*sli* null allele) [10, 24, 25], *robo*^*570*^ (*robo* null allele) [8, 9] (gift of G. Bashaw), *robo2*^*x135*^ (*robo2* null allele) [19] (gift of G. Bashaw), *UAS-sli*^*RNAi*^ (TRiP.JF01228) (Bloomington Drosophila Stock Center (BDSC), Indiana University, IN, BL-31467), the ubiquitous *Act5C*-GAL4; *UAS-dcr2* (BDSC), 69B-GAL4 (BDSC), *UAS-secGFP* (gift of D. Andrew). *sli*^*2*^, *robo*^*570*^, and *robo2*^*x135*^ were balanced over an *SM6 evelacZ* balancer on the second chromosome. β-Galactosidase (β-Gal) was used to mark balancer chromosomes, and mutants were distinguished by an absence of β-Gal. All embryos were collected at 25 °C.

### Staining procedures

HRP and fluorescence immunohistochemistry were performed as described previously [26–28]. The following primary antibodies were used: rabbit anti-β-gal (1:3000, Molecular Probes), mouse anti-22C10 (1:10, Developmental Studies Hybridoma Bank (DSHB), University of Iowa, IA), rat anti-Elav (7E8A10) (1:10, DSHB), mouse anti-Repo (1:100, DSHB), mouse anti-Prospero (1:10, DSHB), mouse anti-Slit (C555.6D) (1:10, DSHB), mouse anti-Robo (13C9) (1:10, DSHB), rabbit anti-α-tubulin 85E (1:50, Thermo Fisher Scientific), and rabbit anti-GFP (1:5000, ThermoFisher Scientific). The following secondary antibodies were used at 1:500: anti-Mouse 555, anti-Rabbit 488, anti-Rat 488, anti-Rat 647, biotinylated anti-mouse and biotinylated anti-rabbit.

### Imaging

A Nikon Eclipse CiL compound light microscope using differential interference contrast (DIC) optics was used to take images of Horseradish Peroxidase- (HRP)-stained embryos at 20X and 40X magnification. A Nikon Eclipse Ti Confocal Microscope was used to image fluorescence-stained embryos at 40X and 60X objective lenses. All the images were analyzed and processed using Nikon Elements Ar software.

### Statistical analysis

A G-test of statistical independence was used to compare abnormality in lch5 neuron migration patterns as well as the classes of defects and the percentages.

## Results

### Absence of *sli* displays improper migration and irregular morphology of lch5 neurons

**Fig. 2:**
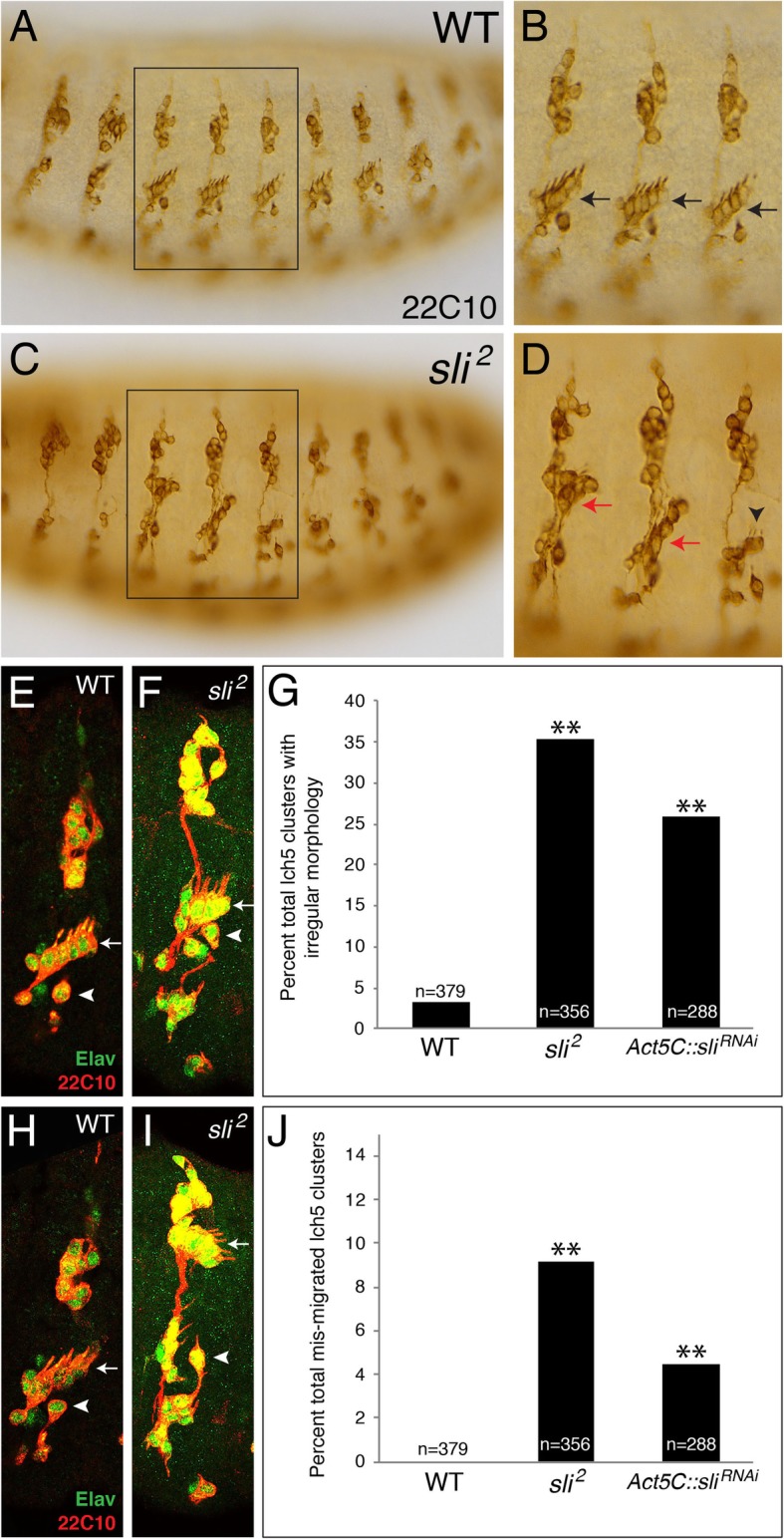
Absence of *sli* results in mis-migrating and morphological irregularities in lch5 chordotonal neurons. **a**. WT stage 16 embryo labeled with anti-22C10 to mark all PNS sensory neurons showing the lch5 cluster in a lateral position in the embryo and the five lch5 neurons in an evenly-spaced diagonal row facing the dorsal-posterior edge of the embryo. **b**. Blown-up view of boxed region in A showing three abdominal hemisegments of PNS neurons. Black arrows point to lch5 neurons. **c**. *sli* mutant (*sli*^*2*^) embryo showing lch5 neurons either mis-migrating or displaying an irregular morphology. **d**. Blown-up view of boxed region in C showing three abdominal hemisegments of PNS neurons. Red arrows show lch5 neurons that have mis-migrated, and black arrowhead shows lch5 neurons with an irregular morphology. **e**. One hemisegment of a WT embryo labeled with anti-22C10 (red) which marks the cell membranes of all PNS neurons and anti-Elav (green) which stains the nuclei of all neurons. White arrow points to the lch5 neurons with normal morphology and the white arrowhead points to the v’ch1 neuron which is normally just ventral to the lch5 cluster. **f**. *sli*^*2*^ embryo showing lch5 neurons with irregular morphology (white arrow). The white arrowhead points to the v’ch1 neuron which is normally just ventral to the lch5 cluster. **g**. Quantification of percent total lch5 clusters displaying irregular morphology in WT, *sli*^*2*^, and a ubiquitous knock-down of *sli* using RNAi (*Act5C*-GAL4::*UAS-sli*^*RNAi*^) abbreviated as *Act5C*::*sli*^*RNAi*^. **h**. WT embryo showing lch5 cluster that has migrated to the correct lateral position. White arrow points to the position of the lch5 neurons and the white arrowhead points to the v’ch1 neuron which is normally just ventral to the lch5 cluster. **i**. *sli*^*2*^ embryo showing lch5 neurons that have not migrated ventrally (white arrow). The white arrowhead points to the v’ch1 neuron. The increased distance between the lch5 neurons and the v’ch1 neurons is visualized in the *sli*^*2*^ compared to the WT in **h**. **j**. Quantification of percent total lch5 mis-migrating clusters in WT, *sli*^*2*^, and *Act5C*::*sli*^*RNAi*^. Number of abdominal lch5 clusters scored for each genotype above each bar (n). **p < 0.05 based on a G-test of statistical independence

It was mentioned previously, but not shown, that the absence of *sli* resulted in mis- or non-migration of the lch5 chordotonal organs [19]. Therefore, we wanted to examine more closely the lch5 neurons in embryos missing the extracellular ligand *sli*. Our experiments show that indeed, while wild-type embryos displayed lch5 neurons with a teardrop shape and dendrites facing dorsal-posterior (Fig. 2A, B, black arrows), the absence of *sli* (*sli*^*2*^) showed two striking defects: irregular morphology of the lch5 neurons (Fig. 2C, D, black arrowhead) and mis-migrating lch5 neurons (Fig. 2C, D, red arrows). We defined irregular morphology as the lch5 neurons displaying the following traits: neurons lacking a teardrop shape, neurons not slightly overlapping, and dendrites pointing in aberrant directions instead of dorsal-posterior (compare Fig. 2F to Fig. 2E). When quantified, *sli* mutants displayed 30% more lch5 neurons with an irregular morphology compared to wild-type (Fig. 2G).

In the wild-type, the lch5 organ migrates ventrally to its final position in the lateral cluster of peripheral neurons [15, 17, 18]. We found that *sli* mutants displayed mis-migrating lch5 neurons that either do not migrate as a cluster and end up in various positions along the dorsal-ventral axis (Fig. 2C, D, red arrows) or completely fail to migrate (Fig. 2I, white arrow). This failure to migrate can be seen by the distance between the lch5 cluster and the v’ch1 neuron (compare white arrow (lch5 cluster) to white arrowhead (v’ch1 neuron) in Fig. 2H and Fig. 2I). When quantified, *sli* mutants displayed a significantly greater number of lch5 neurons that mis-migrated or failed to migrate ventrally to their target location (Fig. 2J). In order to confirm that both these defects were the result of an absence of *sli*, we also knocked down *sli* using a ubiquitous GAL4 driver, *Act5C*-GAL4 along with *UAS-dcr2* (dicer2) to facilitate the RNA interference (RNAi). Surprisingly, knocking down *sli* displayed a similar, but less severe lch5 neuronal defects than the *sli*^*2*^ null allele (Fig. 2G and J). We interpret this to mean that even a small amount of Sli protein present can prevent the lch5 neuronal defects. Clearly, these data suggest that Sli secreted into the ECM surrounding these PNS neurons plays an important role in their migration and final patterning. We next wanted to know whether two Sli receptors, Robo and Robo2, play a role in lch5 neuronal morphology and migration.

### Sli is expressed in the epidermis while Robo is expressed on lch5 neurons

**Fig. 3:**
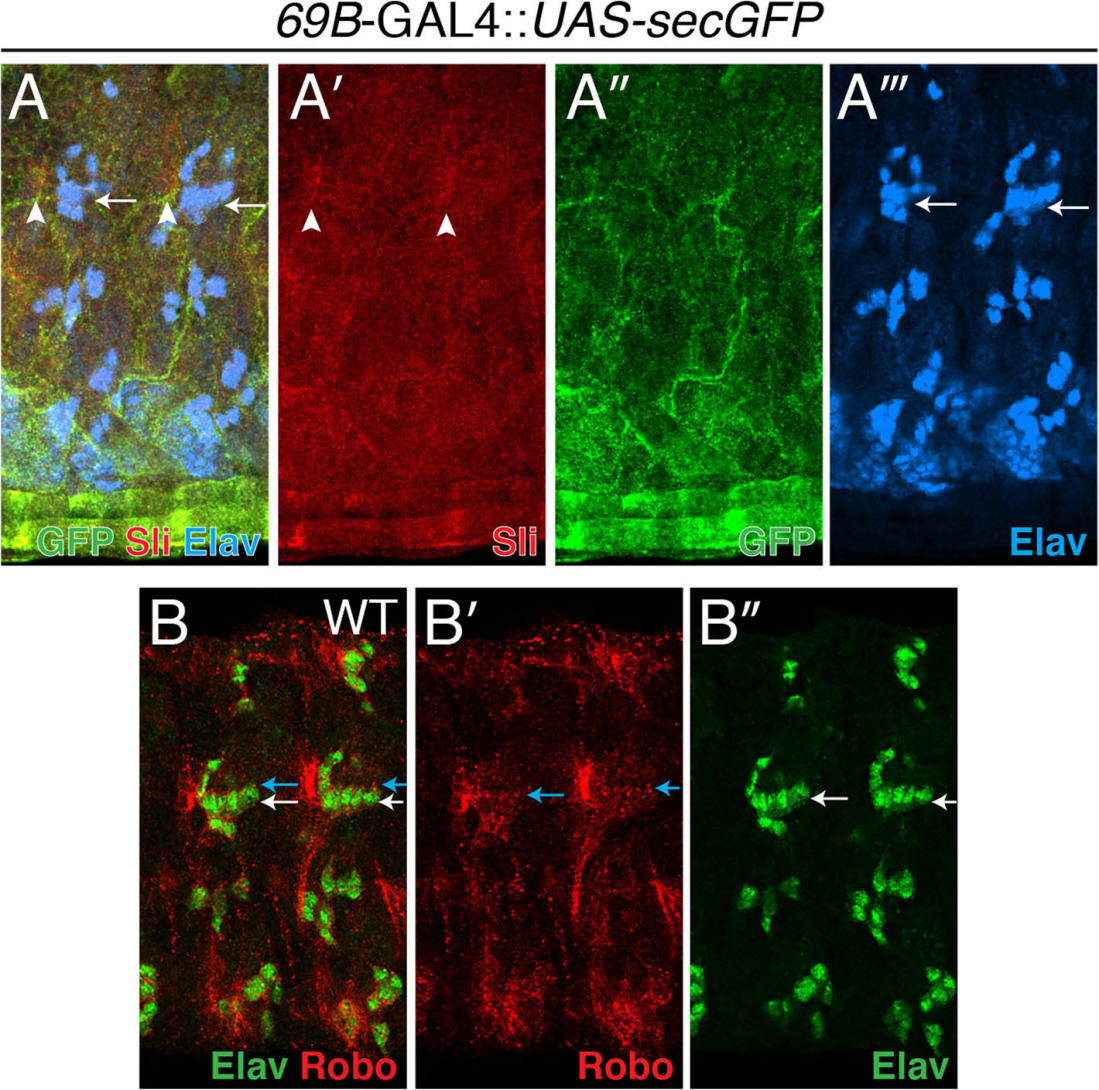
Sli is expressed in the epidermis while Robo is expressed on lch5 dendrites. A-A”’. Epidermal *69B*-GAL4 was used to drive a secreted GFP (*UAS-secGFP*) (*69B*-GAL4:: *UAS-secGFP*) and labeled with anti-Sli (red) (A’), anti-GFP (green) (A”), and anti-Elav (blue) (A”’). White arrows point to lch5 neurons (A and A”’) and white arrowheads point to muscle attachment sites (MASs) expressing Sli (A and A’). B-B″. WT embryo labeled with anti-Robo (red) (B′) and anti-Elav (green) (B″). White arrows point to lch5 neurons and blue arrows point to lch5 dendrites expressing Robo

Before examining *robo* and *robo2* mutants for potential lch5 defects, we wanted to confirm the expression patterns of Sli and Robo. It has been shown that Sli protein gets secreted from epidermal cells [17, 19]. We wanted to examine more closely the expression of Sli in relation to the lch5 neurons. Therefore, we used an epidermal-specific GAL4 driver (*69B*-GAL4) to express a secreted GFP (*UAS-secGFP*) and compare the expression of the secreted GFP to Sli, and compare Sli to the lch5 neurons (Fig. 3A-A”’). Sli expression (red) overlaps with the GFP secreted from the epidermal cells (green) (compare Fig. 3A’ to 3A”) and is not expressed in the lch5 neurons (blue) (white arrows in Fig. 3A and A”’). It has previously been shown that Robo is expressed at the tips of the lch5 dendrites [17]. Here we confirm that Robo (red) is indeed expressed at the tips of the lch5 dendrites (Fig. 3A, B, blue arrows), as well as faintly along the membrane of the lch5 neuronal cell bodies (green) (Fig. 3A, C, white arrows). These results suggest the possibility that Robo acts as the receptor for Sli in the migration and positioning of the lch5 chordotonal neurons.

### Absence of *robo* or *robo2* result in mis-migrating and morphological irregularities in lch5 chordotonal neurons

**Fig. 4:**
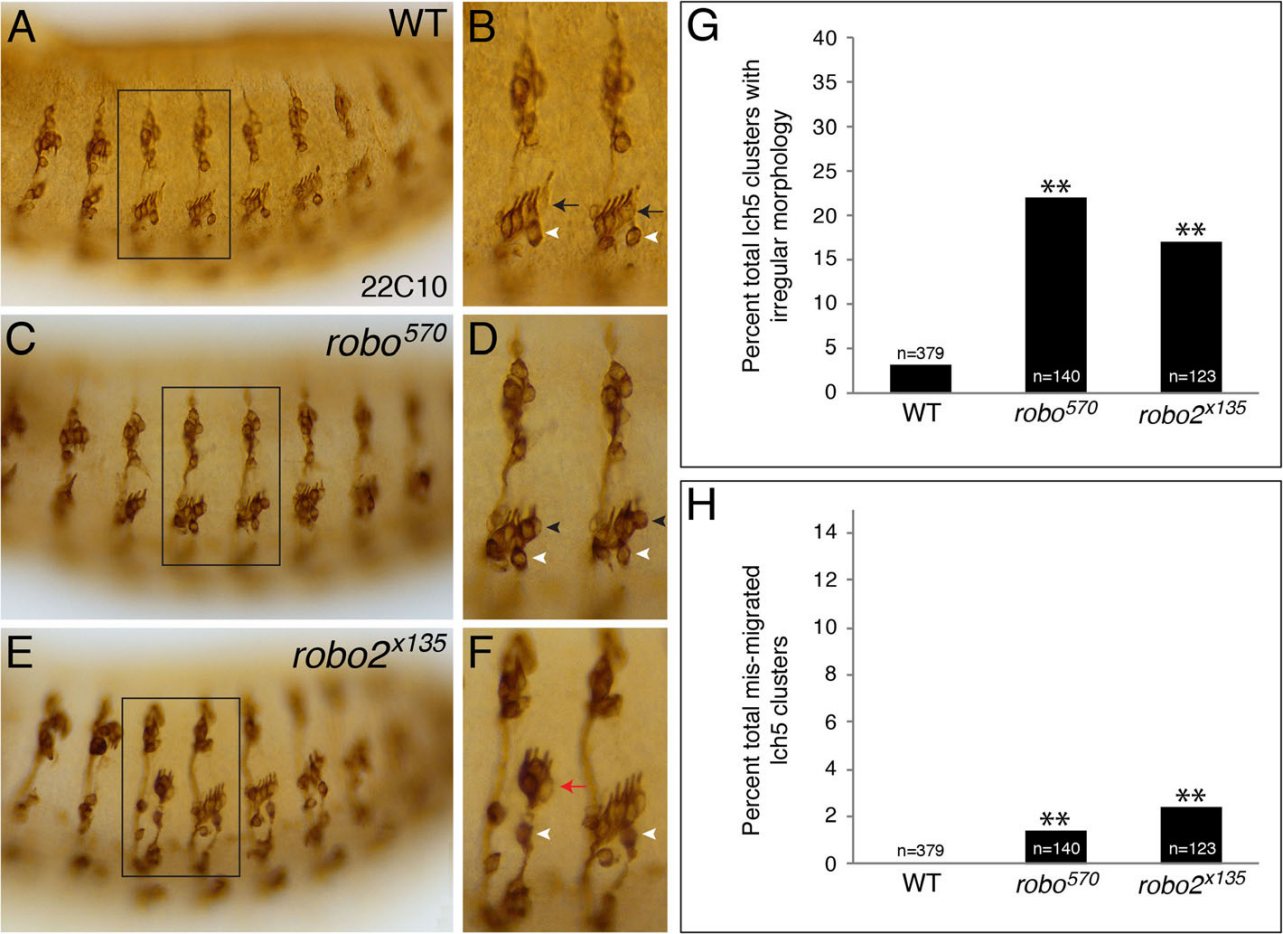
Absence of *robo* or *robo2* result in mis-migrating and morphological irregularities in lch5 chordotonal neurons. **a**. WT stage 16 embryo labeled with anti-22C10 showing the lch5 cluster in a lateral position in the embryo and the five lch5 neurons in an evenly-spaced diagonal row facing the dorsal-posterior edge of the embryo. **b**. Blown-up view of boxed region in A showing two abdominal hemisegments of PNS neurons. Black arrows point to lch5 neurons and white arrowheads point to the v’ch1 neuron, just ventral to the lch5 cluster. **c**. *robo* mutant (*robo*^*570*^) embryo showing lch5 neurons displaying an irregular morphology. **d**. Blown-up view of boxed region in C showing two abdominal hemisegments of PNS neurons. Black arrowheads show lch5 neurons with an irregular morphology and white arrowheads point to the v’ch1 neuron. **e**. *robo2* mutant (*robo2*^*x135*^) embryo showing lch5 neurons not migrating as far ventral. **f**. Blown-up view of boxed region in E showing two abdominal hemisegments of PNS neurons. Red arrow shows lch5 neurons that are further from the v’ch1 neuron (white arrowhead). **g**. Quantification of percent total lch5 clusters displaying irregular morphology in WT, *robo*^*570*^, and *robo2*^*x135*^. **h**. Quantification of percent total mis-migrating lch5 clusters in WT, *robo*^*570*^, and *robo2*^*x135*^. Number of abdominal lch5 clusters scored for each genotype above each bar (n). **p < 0.05 based on a G-test of statistical independence

Based on the reciprocal expression patterns of Sli secreted from the epidermal cells and Robo on the lch5 neurons, we wanted to know whether the absence of *robo* would display any lch5 neuronal defects similar to those observed in the absence of *sli*. Embryos mutant for *robo* (*robo*^*570*^) displayed a significant increase in lch5 neurons with an irregular morphology (Fig. 4C, D, G, black arrowheads in 4D). The absence of *robo* also displayed a slight, but significant, increase in mis-migrating lch5 neurons (Fig. 4H). Interestingly, embryos mutant for *robo2* (*robo2*^*x135*^) also displayed a significant increase in lch5 neurons with an irregular morphology (Fig. 4G). Surprisingly, the absence of *robo2* displayed almost twice as many mis-migrating lch5 neurons as *robo* mutants (Fig. 4E, F, H, red arrow in 4F). In this case, the lch5 neurons are too far dorsal as compared to the v’ch1 neuron (white arrowhead in Fig. 4F). These results, as well as previous work, suggests that Robo and Robo2 act together as receptors for Sli to guide and properly position the lch5 chordotonal neurons [19].

### Attachment and cap cells follow aberrant lch5 dendrites in absence of *sli*

**Fig. 5:**
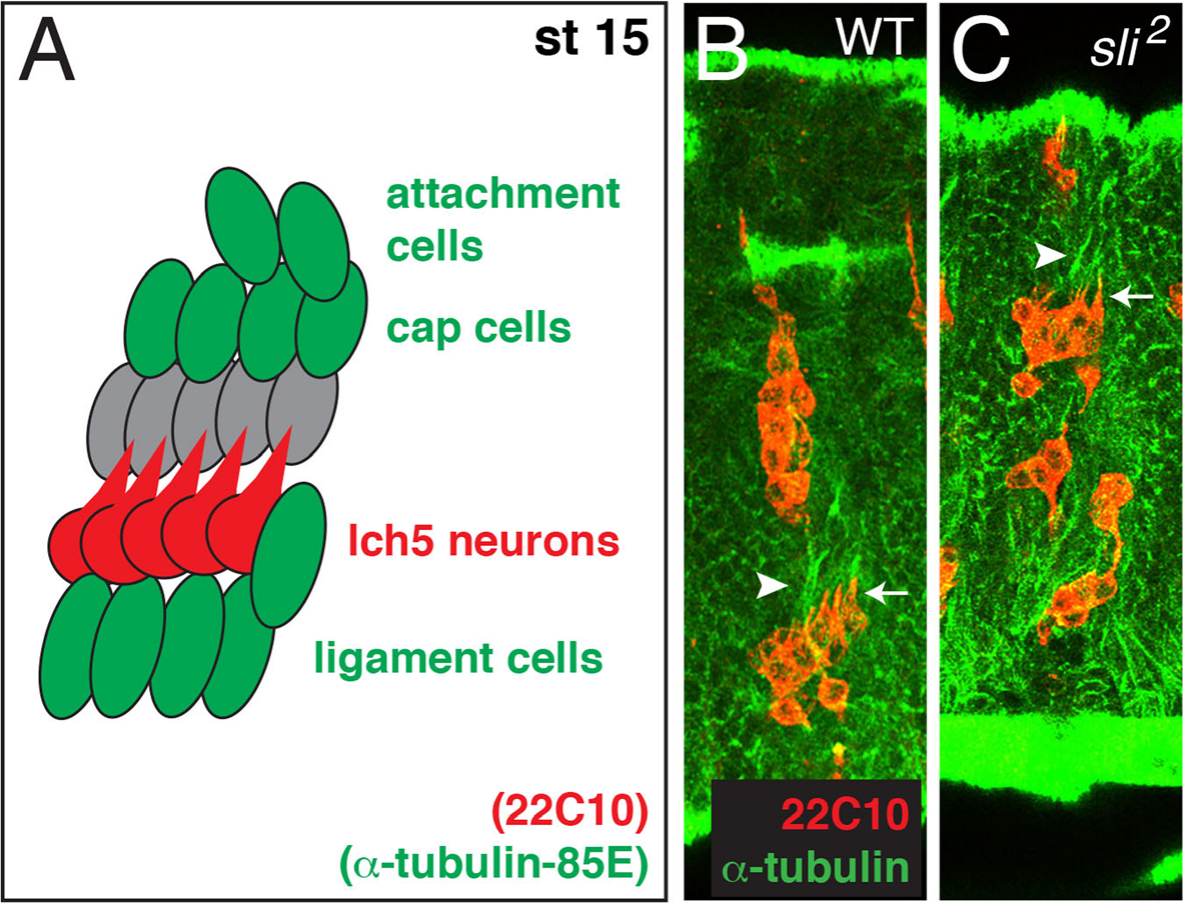
Attachment and cap cells follow aberrant lch5 dendrites in absence of *sli*. **a**. Cartoon depiction of lch5 neurons (red) and three different support cells (green): ligament cells, cap cells, and attachment cells. All embryos labeled with 22C10 (red) to mark all PNS neurons and α-tubulin85E (green) to mark ligament, cap and attachment cells (**b**, **c**). B. WT embryo displaying normal morphology of lch5 neurons. White arrow points to the lch5 neuronal dendrites and white arrowhead points to cap/attachment cells dorsal to the lch5 neurons that appear to be in straight streaks stemming from the lch5 dendrites. **c**. *sli*^*2*^ embryo displaying irregular morphology with dendrites pointing in all directions (white arrow). The cap/attachment cells dorsal to the lch5 cluster (white arrowhead) follow the aberrant dendrites stemming from the lch5 neurons. The total number of abdominal lch5 clusters examined: WT (*n* = 34), *sli*^*2*^ (*n* = 28)

Knowing that the lch5 chordotonal neurons are surrounded by several different types of glial cells, we wanted to know how loss of *sli* affects the position of these glial cells. The ligament cells, cap cells, and attachment cells all express the molecular marker α-tubulin85E [18], shown in a cartoon diagram (Fig. 5A, green cells) compared to the lch5 neurons (Fig. 5A, red cells). Of these specific glial cells, only the ligament cells come into direct contact with the lch5 neurons (Fig. 5A, ventral green cells) [18, 22]. Although the ligament cells were harder to visualize with α-tubulin85E, we did observe tubulin filaments (Fig. 5B, white arrowhead) dorsal to the lch5 neurons pointing in the same direction as the dendrites in wild-type embryos (Fig. 5B, white arrow). In *sli* mutants when the lch5 neurons have mis-migrated or displayed an irregular morphology, tubulin filaments (Fig. 5C, white arrowhead) are seen pointing in the same direction as the irregularly pointed dendrites (Fig. 5C, white arrow). These data suggest that the support cells dorsal to the lch5 neurons, the cap and attachment cells, follow the irregularly pointed dendrites on the lch5 neurons in the absence of *sli*.

### Scolopale cells follow mis-migrating lch5 neurons in absence of *sli*

**Fig. 6:**
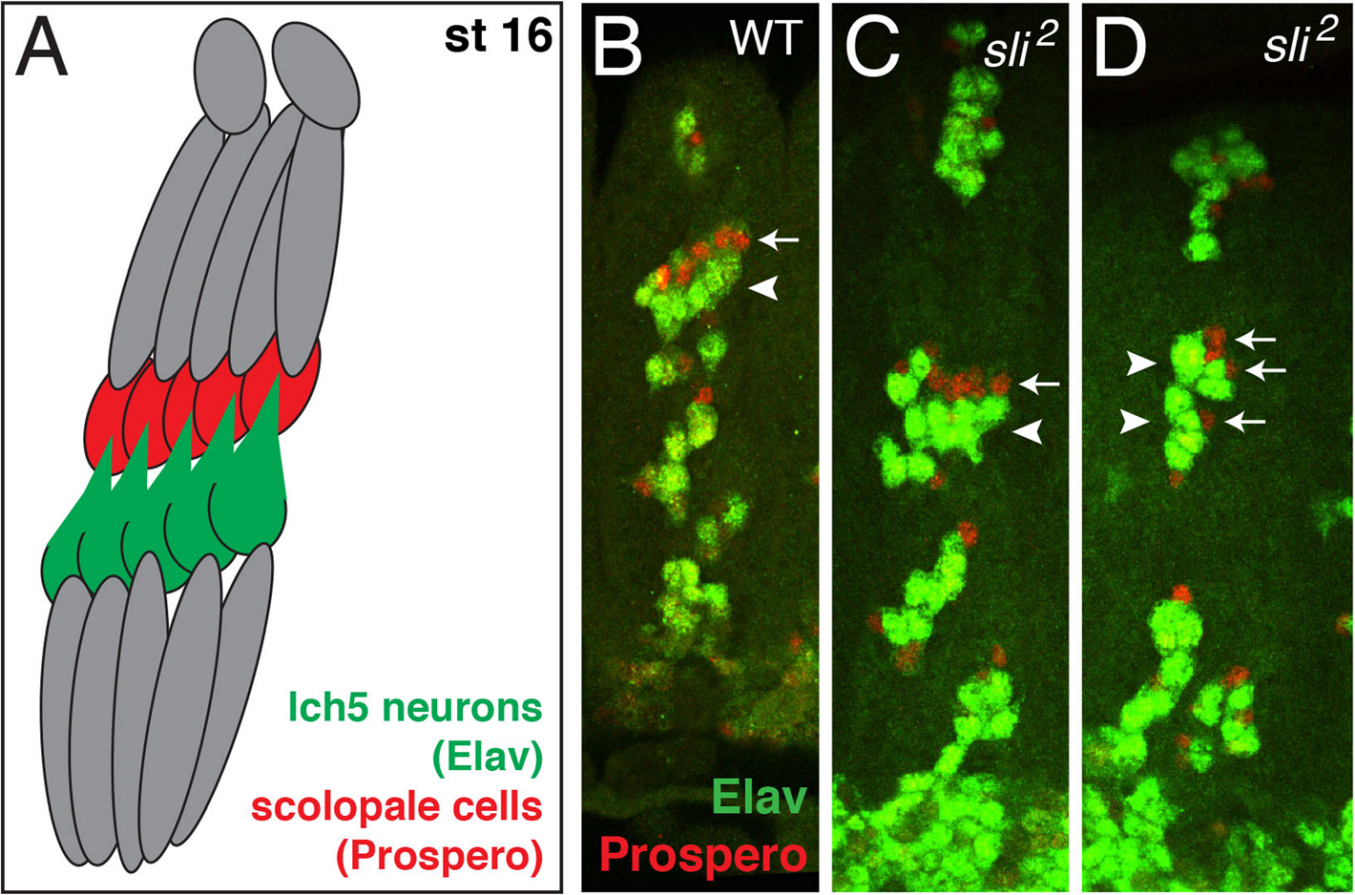
Scolopale cells follow mis-migrating lch5 neurons in absence of *sli*. **a**. Cartoon depiction of stage 16 lch5 chordotonal organs with lch5 neurons (green) and scolopale cells (red). All embryos labeled with anti-Elav (green) to mark lch5 neurons and anti-Prospero (red) to mark scolopale cells (**b**-**d**). **b**. WT embryo showing five scolopale cells (white arrow) directly dorsal and associating with the five lch5 neurons (white arrowhead). **c**. *sli*^*2*^ embryo showing lch5 neurons in irregular positions (white arrowhead) and the five scolopale cells (white arrow) still associating with and dorsal to the lch5 neurons (white arrowhead). **d**. *sli*^*2*^ embryo where the lch5 neurons have not migrated ventrally (white arrowheads), each lch5 neuron is still associated with a scolopale cell (white arrows). The total number of abdominal lch5 clusters examined: WT (*n* = 46), *sli*^*2*^ (*n* = 30)

The scolopale cells are glial cells that are dorsal to the lch5 neurons and in direct contact with the dendrites of the lch5 neurons (Fig. 6A, scolopale cell in red, lch5 neurons in green). We have already seen that the cap and attachment cells (glial cells that are also dorsal to the lch5 neurons) follow aberrantly pointing dendrites of the lch5 neurons in the absence of *sli*, we asked whether the scolopale cells are also following the mis-migrating lch5 neurons in the absence of *sli*. To answer this question, we used a marker specific for scolopale cell nuclei, Prospero. In wild-type, the scolopale cells are dorsal to the lch5 neurons (Fig. 6B, white arrow). For this experiment we labeled the lch5 neurons with anti-Elav (a pan-neuronal nuclear marker), and therefore, were not able to visualize the lch5 dendrites. However, in the absence of *sli* the lch5 neurons are not positioned properly in relation to each other (compare the lch5 neurons in Fig. 6C, white arrowhead with lch5 neurons in 6B, white arrowhead), and the scolopale cells are still dorsal to these neurons (Fig. 6C, white arrow). Additionally, when the lch5 neurons have not migrated fully, the scolopale cells continue to associate with the lch5 dendrites (Fig. 6D, white arrows). These data suggest that the scolopale cells also follow the mis-migrating and irregularly shaped lch5 neurons in the absence of *sli*.

### Ligament cells continue stretching ventrally in the absence of *sli*

**Fig. 7:**
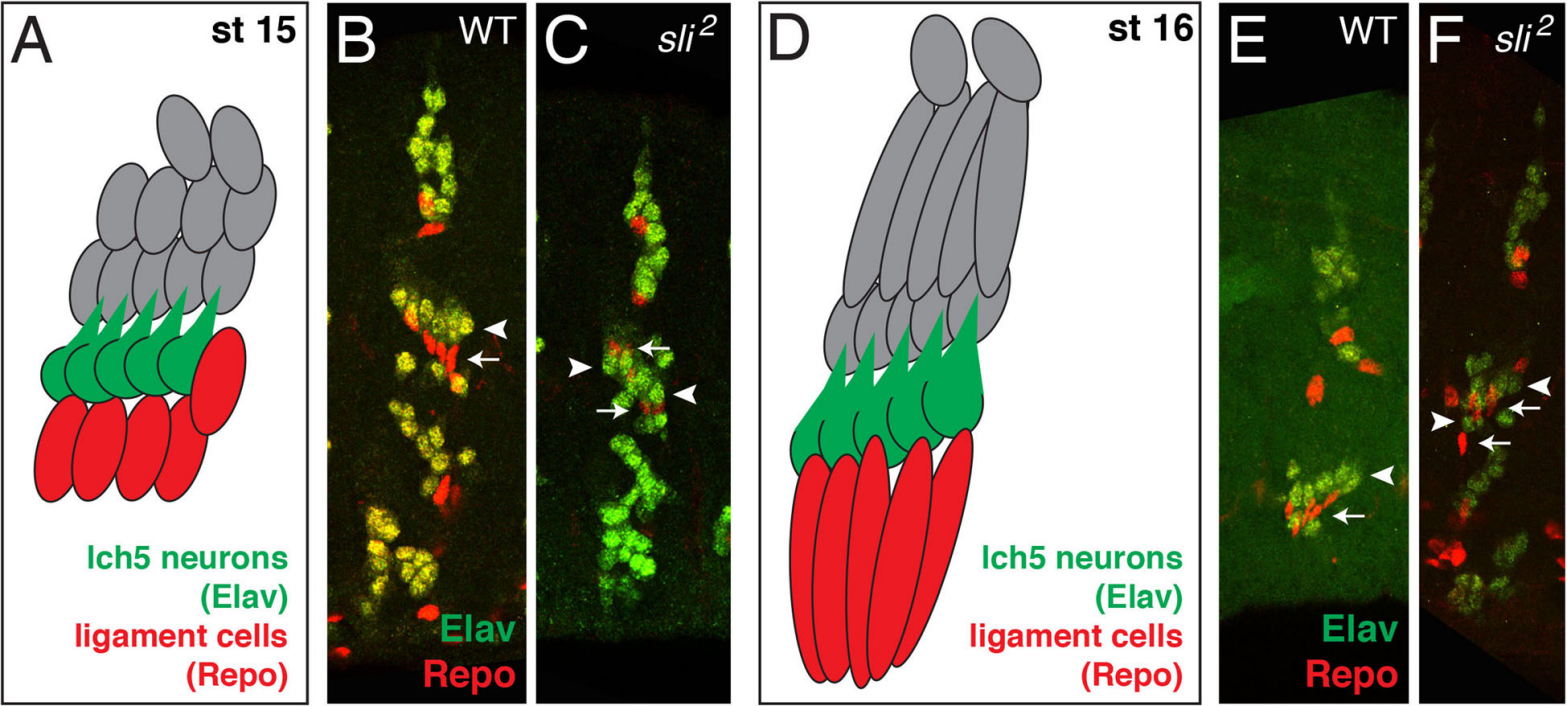
Ligament cells continue stretching ventrally in absence of *sli*. **a**. Cartoon depiction of stage 15 chordotonal organs with lch5 neurons (green) and ligament cells (red) located ventral to the lch5 neurons. All embryos labeled with anti-Elav (green) to mark lch5 neurons and anti-Repo (red) to mark ligament cells (**b**, **c** and **e**, **f**). **b**. Stage 15 WT embryo showing ligament cells (white arrow) directly ventral to the lch5 neurons (white arrowhead). **c**. *sli*^*2*^ embryo showing lch5 neurons in irregular positions (white arrowheads) and ligament cells adjacent to the lch5 neurons, either dorsal or ventral to the lch5 neurons (white arrows). **d**. Cartoon depiction of stage 16 lch5 chordotonal organs with lch5 neurons (green) and ligament cells (red) stretched ventrally. **e**. Stage 16 WT embryo showing the lch5 neurons in their normal position facing dorsal-posterior (white arrowhead), and the ligament cells elongating and stretching ventrally (white arrows). **f**. *sli*^*2*^ embryo showing the lch5 neurons in irregular positions (white arrowheads) and the ligament cells still in their proper position ventral to the lch5 neurons stretching ventrally (white arrows). The total number of abdominal lch5 clusters examined: WT (*n* = 45), *sli*^*2*^ (*n* = 33)

The ligament cells are ventral to the lch5 neurons and stretch ventrally from stage 15 (Fig. 7A, red cells) to stage 16 (Fig. 7D, red cells) [18, 22]. We asked whether the ligament cells are coordinated with the lch5 neurons, even when they have mis-migrated or do not orient themselves correctly, as seen in the absence of *sli* (Fig. 2). At stage 15, wild-type ligament cells can be seen directly ventral to the lch5 neurons (Fig. 7B, white arrow). However, in *sli* mutants where the lch5 neurons are not in the normal orientation, the ligament cells are either ventral or dorsal to the lch5 neurons (Fig. 7C, white arrows). It is not possible to know whether the ligament cells are dorsal or ventral to the lch5 neurons because we are using a nuclear marker for the neurons and, therefore, cannot see where the dendrites of the lch5 neurons are compared to the ligament cells. However, we have never observed an lch5 neuron with its dendrite pointing ventrally. At stage 16, the ligament cells in wild-type start stretching ventrally and have a more elongated shape (Fig. 7E, white arrow). Interestingly, the same stretching of the ligament cells ventrally with a more elongated shape is seen in *sli* mutants (Fig. 7F, white arrow). These data suggest that the ventral stretching of the ligament cells is independent of mis-migrating lch5 neurons.

## Discussion

In this study we show that the extracellular ligand *sli* is required for the ventral migration and morphology of the lch5 chordotonal neurons in the Drosophila embryonic PNS (Fig. 2). The absence of *sli* results in lch5 neurons that do not migrate ventrally enough or that have aberrantly pointing dendrites. Interestingly, the absence of the Sli receptors *robo* and *robo2* display similar lch5 neuronal defects to that of *sli* mutants (Fig. 4), which adds another role to Slit-Robo signaling in neural development. Moreover, we examined the glial support cells that surround the lch5 neurons and make up the lch5 organ. We found that the dorsal glial cells (scolopale, cap, and attachment cells) followed the mis-migrated neurons in the absence of *sli* (Figs. 5, 6). Conversely, the ligament cells, which are ventral to the lch5 neurons, seem to be independent of the lch5 neurons, as they continue stretching ventrally even when the lch5 neurons mis-migrated (Fig. 7). These results provide further evidence for the possible role for Slit-Robo signaling in lch5 neuronal migration and positioning.

### Slit-Robo signaling in the PNS

The Slit-Robo signaling pathway plays a critical role in the development of several organ systems, either as repulsion or attraction [5, 10, 11, 17, 19, 29–32]. In the CNS, Sli is secreted by the midline glia, and the absence of Slit-Robo signaling results in longitudinal axons over-migrating by re-crossing the midline due to a complete lack of repulsive signaling [8–11, 33]. In the embryonic PNS, Sli is expressed in the ectoderm [19] as well as in the visceral mesoderm [17], and secreted into the extracellular environment to interact with various Robo and Netrin receptors to guide neurons and axons [17, 19, 34]. In the thoracic segments of the PNS, the absence of Slit-Robo signaling resulted in thoracic chordotonal neurons migrating ventrally when they normally do not migrate, meaning that Slit-Robo signaling normally inhibits thoracic chordotonal neuron migration [17]. However, in this study we focused on the abdominal segments, and in the absence of *sli*, the abdominal chordotonal neurons (lch5) did not migrate ventrally. This result could mean that Sli is acting as a repellant, same as in the thoracic segments. The expression of Robo and Robo2 on the dendrites of the lch5 neurons [17, this study] could mean that the interaction between the Robo receptors on the lch5 neurons and Sli in the extracellular space dorsal to the lch5 neurons results in the neurons migrating away from Sli in the ventral direction. The absence of either Robo receptor alone displays a defect similar, but less severe, to *sli* mutants. Therefore, a double mutant of *robo* and *robo2* could show a much closer defect to *sli* mutants, as has been mentioned [19]. If Robo and Robo2 were not expressed on the dendrites that are facing dorsal, it might be a possibility that Slit-Robo signaling might be acting as an attraction, as in the somatic muscles [29].

Although a significant number of lch5 neurons mis-migrate, the more prevalent defect we observed was an irregular morphology, namely aberrantly pointing dendrites in the absence of Slit-Robo signaling (Figs. 2 and 4). Two possibilities arise as an explanation for this defect: (1) if Sli is not present, Robo and Robo2 have no attractive signal to help the lch5 neurons position themselves properly, or (2) if Sli is not present, Robo and Robo2 have no repulsive signal to stay away from, and as a result, the dendrites are pointing in all directions. Both possibilities are plausible, however, the mis-migration defect leans toward Slit-Robo signaling acting in a repulsive way. Over-expression analysis of *sli*, *robo* and *robo2* would need to be done to figure out the exact mechanism of *sli* action in the migration and rotation of the lch5 neurons.

### Dorsal glial cells and the lch5 chordotonal organ

In the absence of *sli*, the dorsal secondary support cells, scolopale, cap, and attachment cells, seem to follow the mis-migrating lch5 neurons and aberrantly pointing dendrites (Figs. 5 and 6), which brings up the question of the role of these particular glial cells in the final formation of the lch5 chordotonal organs. If these dorsal glial cells were independent of the lch5 neurons and responded to other signals, we might expect these glial cells to migrate and position themselves correctly in *sli* mutants. However, there may be signals coming from the lch5 dendrites that signal to the scolopale, cap, and attachment cells to stay with or follow the neurons, even if the neurons do not migrate or position themselves properly. It would be interesting to examine these glial cells in other mutants where the lch5 neurons mis-migrate. A recent report in *C. elegans* describes one of the functions of glial cells associated with sensory neurons is to control the shape of the neuronal endings [35]. This study brings up the issue that glial cells are not passive, but instructive, especially in the final positioning and functioning of sensory neurons. Therefore, it would also be interesting to observe lch5 neuron migration and positioning in the absence of the glial cells.

### Ligament cells and ventral migration of lch5 chordotonal organs

Neurons and glial cells function together in development of the nervous system. We, as well as others, have shown when *sli* is missing the ligament cells do not have any attachment to the neurons and continue to stretch ventrally on their own (Fig. 7) [17, 18, 22]. This result does not definitively indicate whether the ligament cells are independent of the lch5 neurons, only that they may also be responding to an attractive cue from the ventral ectoderm. Additionally, the absence of *sli* does not give us the following information: (1) which signal the ligament cells are responding to, (2) whether the signal is attractive or repulsive, and (3) where is the signal coming from. Much more work would need to be done to characterize the ligament cells and what cell surface receptors they express.

## Conclusion

In conclusion, our study suggests an added role for Slit-Robo signaling in the development of the nervous system, namely in migration and positioning of the lch5 chordotonal neurons. Although the defects observed in the absence of Slit-Robo signaling are not severe, they are clear, and point to the idea that several different signaling pathways must function together to allow for proper development of any organ system. Clearly, this study opens the door for additional work on the unique properties of the lch5 organ.

## Data Availability

The data sets used and/or analyzed during the current study are available from the corresponding author on reasonable request.

## Abbreviations

lch5: lateral chordotonal organs
PNS: Peripheral nervous system
*sli*: *slit*
